# Randomized phase-II evaluation of letrozole plus dasatinib in hormone receptor positive metastatic breast cancer patients

**DOI:** 10.1038/s41523-019-0132-8

**Published:** 2019-10-28

**Authors:** Devchand Paul, Svetislava J. Vukelja, Frankie Ann Holmes, Joanne L. Blum, Kristi J. McIntyre, Deborah L. Lindquist, Cynthia R. Osborne, Ines J. Sanchez, Jerome H. Goldschmidt, Yunfei Wang, Lina Asmar, Lewis Strauss, Joyce O’Shaughnessy

**Affiliations:** 1 US Oncology Research, Inc., 10101 Woodloch Forest Dr., The Woodlands, TX 77380 USA; 2 Rocky Mountain Cancer Centers, 4700 East Hale Park Way #400, Denver, CO 80220 USA; 3 Texas Oncology–Tyler, 910 E Houston St #100, Tyler, TX 75702 USA; 4 Texas Oncology–Houston Memorial City, 925 Gessner #550, Houston, TX 77024 USA; 5 Texas Oncology at Baylor University Medical Center, 3410 Worth Street, Dallas, TX 75246 USA; 6 Texas Oncology–Dallas Presbyterian Hospital, 8196 Walnut Hill #100, Dallas, TX 75231 USA; 7 Arizona Oncology Associates, 3700W State Route 89A, Sedona, AZ 86336 USA; 8 Texas Oncology, 1901 Grandview, El Paso, TX 79902 USA; 9 Blue Ridge Cancer Care, 2600 Research Center Drive, Suite A, Blacksburg, VA 24060 USA; 10 Bristol-Myers Squibb, 5 Research Pkwy, Wallingford, CT 06492 USA

**Keywords:** Cancer therapy, Breast cancer

## Abstract

The non-receptor tyrosine kinase Src activation plays a role in the malignant progression of breast cancer, including development of endocrine therapy resistance and survival of bone metastases. This study investigated whether adding Src kinase inhibitor dasatinib to aromatase inhibitor (AI) therapy improved outcomes in estrogen receptor (ER)-positive, HER2-negative metastatic breast cancer (MBC). Postmenopausal patients with ER-positive, HER2-negative MBC (0–1 prior chemotherapies and no prior AI for MBC) were eligible for this non-comparative, parallel group, phase-II study. Patients were randomized to letrozole (2.5 mg/day PO) alone or with dasatinib (100 mg/day PO). Patients with disease progression on letrozole alone could crossover to dasatinib plus continued letrozole. The primary endpoint was clinical-benefit-rate (CBR; complete response + partial response + stable disease ≥6 months). A total of 120 patients were randomized. The CBR of 71% (95% CI 58–83%) was observed with letrozole + dasatinib versus the projected CBR of the combination of 56%. The CBR of 66% (95% CI 52–77%) with letrozole alone also exceeded the projected CBR of 39% with letrozole alone. The CBR was 23% in the crossover arm of letrozole plus dasatinib in patients progressing on letrozole alone. Median progression-free survival with the combination was 20.1 months and 9.9 months with letrozole alone. Letrozole plus dasatinib was well tolerated, although 26% of patients required dasatinib dose reductions. In this non-comparative phase-II trial, the CBR of 71% and the median PFS of 20.1 months with letrozole + dasatinib are encouraging and suggest that dasatinib may inhibit the emergence of acquired resistance to AI therapy.

## Introduction

Src is a pleiotropic, membrane-associated, non-receptor tyrosine kinase that has been implicated in the proliferation, survival, migration, and invasion of breast cancer cells, as well as the development of resistance to therapy.^1–3^ In vitro studies have shown that Src interacts with a variety of signaling molecules including growth factor receptors, integrins, and steroid hormone receptors.^1–3^ In breast cancer cells, membrane-associated estrogen receptor-alpha (ERα) has been shown to complex with Src and phosphatidylinositol 3-kinase (PI3K) to drive growth and endocrine therapy resistance.^4–6^ Levels of ERα and Src are inversely correlated in primary breast cancers, and Src has been shown to increase the proteolytic degradation of Erα.^7^ Combining Src inhibitors with endocrine agents can reduce the emergence of acquired endocrine resistance in preclinical studies.^1,8^ Further, in a phase-II clinical study of the Src inhibitor bosutinib as monotherapy in 73 patients with previously treated advanced or metastatic breast cancer (MBC), all four responses occurred in patients with hormone receptor (HR)-positive disease.^9^ Finally, Src is involved in the regulation of osteoclast-mediated bone turnover,^10^ and has been implicated in the survival and outgrowth of breast cancer cells in the bone marrow microenvironment.^2^

Dasatinib is a potent oral tyrosine kinase inhibitor (TKI) with specificity for a number of related kinases including BCR-ABL, Src, c-KIT, platelet-derived growth factor receptor, and TEC family kinases.^11^ Dasatinib has been approved for the treatment of newly diagnosed Philadelphia chromosome-positive (Ph+) chronic myelogenous leukemia (CML) in chronic phase, as well as Ph + CML or acute lymphoblastic leukemia (ALL) resistant to or intolerant of prior imatinib.^11^ In preclinical models of tamoxifen-resistant breast cancer, dasatinib alone or in combination with tamoxifen or letrozole inhibited tumor growth.^3,5^ In a phase-II clinical trial, dasatinib monotherapy (100 mg or 70 mg BID) produced 2 confirmed partial responses (PRs) and 6 disease stabilizations ≥ 16 weeks in 45 evaluable patients with advanced, pretreated HR-positive breast cancer.^12^ Adverse events were similar to those observed in CML, including fatigue, diarrhea, headache, nausea, and pleural effusion. Dasatinib has also demonstrated bone anabolic and anti-resorptive effects in preclinical systems.^13,14^

This randomized non-comparative phase-II study with a concurrent control arm was designed to evaluate the efficacy and safety of dasatinib plus letrozole for postmenopausal patients with ER-positive, HER2-negative MBC, and to determine if this combination could delay the development of endocrine therapy resistance. In addition, correlative exploratory analyses of protein expression by reverse phase protein array (RPPA) and activation patterns were performed to identify potential biomarkers associated with sensitivity or resistance to letrozole or letrozole plus dasatinib.

## Results

### Patient characteristics

**Fig. 1:**
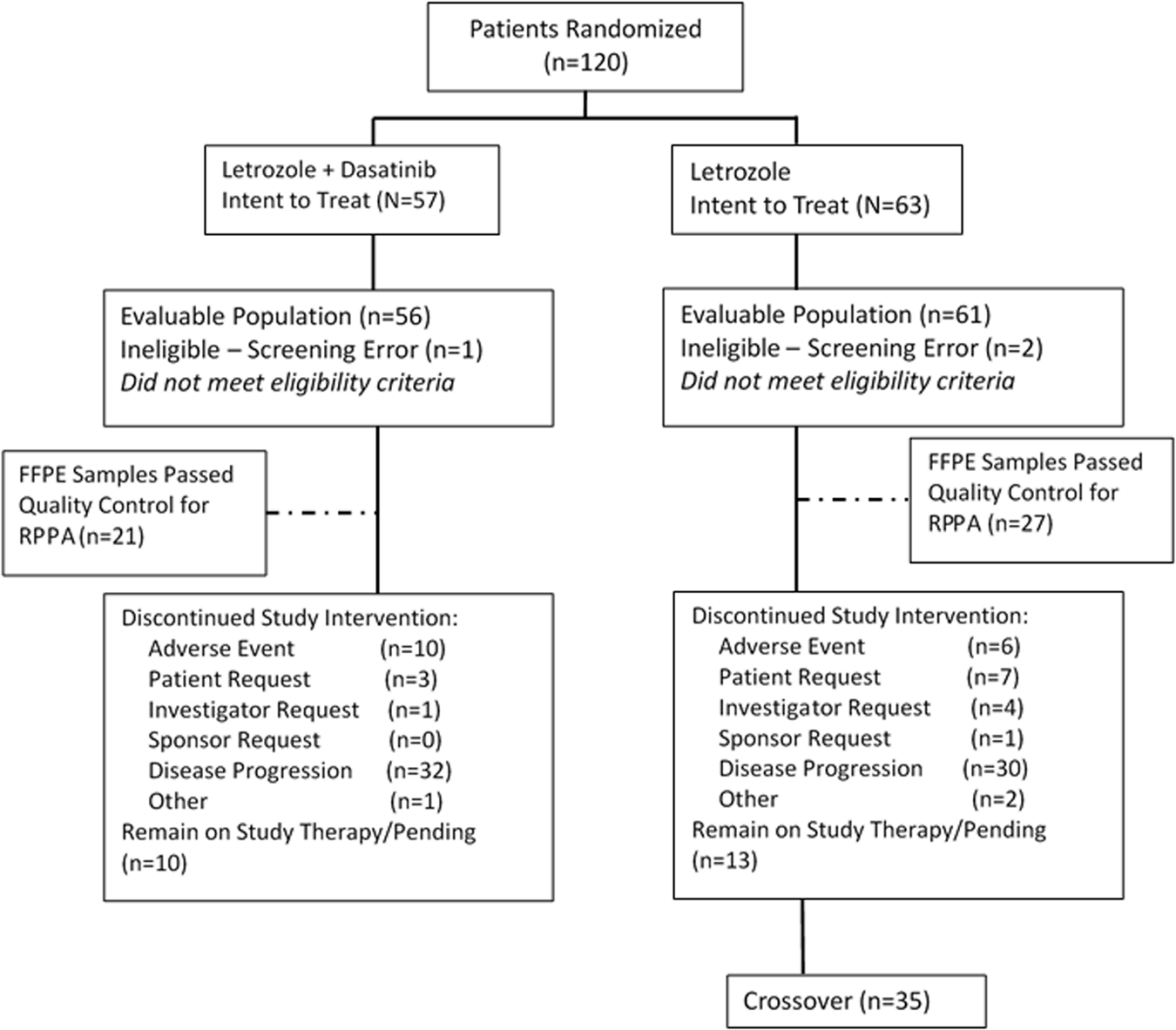
CONSORT diagram. Treatment assignment

A total of 120 patients were enrolled and randomized, 57 to letrozole plus dasatinib and 63 to letrozole monotherapy between 2008 and 2013. (CONSORT diagram—Fig. 1) Patient characteristics were generally well balanced between arms, except that there were more de novo metastatic and endocrine naïve patients in the combination arm (Table 1). Approximately half of patients had received prior chemotherapy, either in the adjuvant or metastatic settings. Approximately half of the patients who had developed recurrent disease had a DFI of ≤2 years.

**Table 1:** Demographics and baseline characteristics (ITT population)

Parameter		Letrozole + Dasatinib (*n* = 57 (%))	Letrozole (*n* = 63 (%))
Age, years (range)	Median	62.3 (37.4, 80.9)	61.3 (35.6, 86.9)
DFI in Recurrent Metastatic Patients			
	<=2 years	27 (47)	34 (54)
	>2 years	30 (53)	29 (46)
	Median (Range) in months	27.5 (0, 277)	21.2 (0, 290)
De Novo stage IV		24 (42)	20 (32)
ECOG performance status	0	33 (58)	40 (64)
	1	24 (42)	22 (35)
	2	0	1 (2)
Histological grade	G1	8 (14)	6 (10)
	G2	22 (39)	28 (44)
	G3	17 (30)	14 (22)
	GX (Grade cannot be assessed)	0	5 (8)
	Unknown	10 (18)	10 (16)
Sites of metastasis	Bone	42 (74)	44 (70)
	Lymph node/soft tissue	18 (32)	18 (29)
	Visceral-liver	10 (18)	12 (19)
	Visceral-lung	8 (14)	10 (16)
Assessment type (evaluable population)	Measurable by RECIST	34 (60)	46 (73)
	Not measurable	22 (39)	15 (24)
	Missing	1 (2)	2 (3)
Prior chemotherapy	Yes	26 (46)	33 (52)
	Adjuvant	22 (39)	30 (48)
	Metastatic	6 (11)	3 (5)
	No	31 (54)	29 (46)
	Unknown	0	1 (2)
Prior endocrine therapy	Yes	22 (39)	32 (51)
	Adjuvant	19 (33)	29 (46)
	Adjuvant-tamoxifen	18 (32)	23 (37)
	Adjuvant-others (aromatase inhibitor)	1 (2)	6 (10)
	Metastatic	4 (7)	3(5)
	Metastatic-tamoxifen	3 (5)	2 (3)
	No	35 (61)	31 (49)
ER|PR status	ER + PR+	53 (93)	57 (90)
	ER+PR−	3 (5)	6 (10)
	ER-PR+	1 (2)	0

### Safety and tolerability

Combination treatment with letrozole plus dasatinib was generally well tolerated, and the majority of adverse events were grade 1/2 (Table 2). The most frequent AEs (all grades) were fatigue (42.1%), nausea (38.6%), rash (26.3%), anemia (24.6%), and neutropenia (22.8%). Grade 3 AEs were uncommon, and included rash and edema (2 events each), and fatigue, anemia, neutropenia, arthralgia, and pleural effusion (1 event each). One patient in the combination arm experienced two grade 4 AEs considered related to study drug: respiratory failure and acute renal failure; both events resolved. Pleural effusion occurred in 9 patients (16%), and all but one were grade 1 or 2 events. No grade 3/4 liver dysfunction was observed. Fifteen patients (26%) in the letrozole plus dasatinib arm required a dasatinib dose reduction. A total of 10 patients (17.5%) in the combination arm and 6 patients (9.5%) in the letrozole alone arm discontinued study treatment due to an AE. Four patients remained on dasatinib plus letrozole for 3 + years.

**Table 2:** Adverse events occurring in ≥10% of patients on study treatment

	Letrozole + Dasatinib (*N* = 57)				Letrozole (*N* = 63)			
Adverse Event	Grade 1 (*n* (%))	Grade 2 (*n* (%))	Grade 3 (*n* (%))	Total (*n* (%))	Grade 1 (*n* (%))	Grade 2 (*n* (%))	Grade 3 (*n* (%))	Total (*n* (%))
Rash	8 (14)	5 (9)	2 (4)	15 (26)	0	0	0	0
Edema	1 (2)	4 (7)	2 (4)	7 (12)	0	0	0	0
Fatigue	15 (26)	8 (14)	1 (2)	24 (42)	3 (5)	2 (3)	0	5 (8)
Anemia	10 (18)	3 (5)	1 (2)	14 (25)	0	0	0	0
Neutropenia	7 (12)	5 (9)	1 (2)	13 (23)	0	0	0	0
Arthralgia	6 (11)	2 (4)	1 (2)	9 (15)	1 (2)	2 (3)	0	3 (5)
Pleural Effusion	4 (7)	4 (7)	1 (2)	9 (16)	0	0	0	0
Hot Flashes	7 (12)	1 (2)	0	8 (14)	10 (16)	2 (3)	1 (2)	13 (20)
Nausea	15 (26)	7 (12)	0	22 (39)	5 (8)	0	0	5 (8)
Diarrhea	7 (12)	3 (5)	0	10 (18)	0	0	0	0
Vomiting	5 (9)	3 (5)	0	8 (14)	0	0	0	0
Headache	5 (9)	2 (4)	0	7 (12)	2 (3)	0	0	2 (3)
Pain	4 (7)	1 (2)	0	5 (9)	4 (6)	4 (6)	0	8 (13)

### Efficacy

The primary endpoint of CBR (CR + PR + SD ≥ 6 months) of this non-comparative trial was the CBR of combined letrozole plus dasatinib which was 71% (95% CI 58–83%), versus the projected CBR of 56% with letrozole plus dasatinib. The CBR observed with letrozole alone was 66% (95% CI 52–77%) which also exceeded the projected CBR of 39% with letrozole alone. The ORRs were 23% (95% CI 13–36%) with letrozole plus dasatinib and 25% (95% CI 15–37%) with letrozole alone in the response-evaluable population. In the subset of 35 patients who crossed over to dasatinib plus letrozole following disease progression on letrozole alone, the CBR was 23% (8/35), with a median PFS of 3.7 months.

**Fig. 2:**
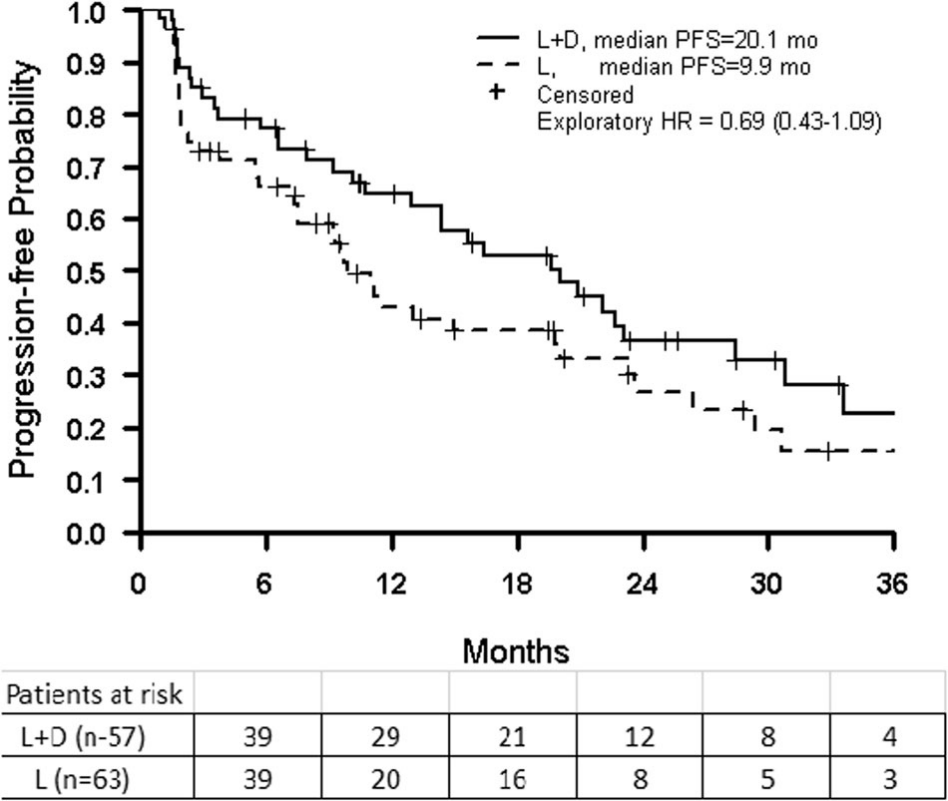
Estimates of progression-free survival in the intent-to-treat population. L letrozole; L + D letrozole plus dasatinib

Median PFS was 20.1 months with the letrozole/dasatinib combination and was 9.9 months with letrozole alone (Fig. 2). Median OS was 48.6 months with the combination and was 41.9 months with letrozole alone.

### Bone mineral density

The percentage of patients with osteopenia, defined in this study as a T score < −1.5 in either the lumbar spine or femur, decreased in patients receiving dasatinib plus letrozole, from 32% at baseline to 14% on study therapy (obtained at least 6 months following initiation of study treatment). There was no change in BMD in patients treated with letrozole alone, with 38% of patients being classified as osteopenic at baseline, and 32% on study therapy. Bisphosphonates therapy was allowed after completion of the first 8 weeks of study treatment; 18 patients on the dasatinib plus letrozole arm received bisphosphonates while on study (1 oral, 17 intravenous), as did 23 patients on the letrozole monotherapy arm (2 oral, 21 intravenous). The duration of bisphosphonate use was not captured.

### Exploratory biomarker analysis

Primary breast cancer FFPE tissues were available for 88 patients, and of these, 48 were adequate for RPPA. Median PFS rates for the 48 patients with adequate tissue for RPPA were very similar to that of the entire study population: 19.2 months for letrozole plus dasatinib (*n* = 21) and 9.2 months for letrozole alone (*n* = 27).

Approximately 90% of the primary breast cancers overexpressed HER3, p-mTOR, p-4EBP1, p-JAK2, p-STAT3, and 50% overexpressed p-Paxillin. Having PFS ≤ 6 months, i.e., no evidence of clinical benefit, was associated with HER3 (*p* < 0.01), p-HER3 (*p* = 0.03), p-Src (*p* = 0.06), p-Paxillin (*p* = 0.02), or p-MET (*p* = 0.05) expression in letrozole monotherapy-treated patients but expression of these phospho-proteins was not associated with poor outcome in the letrozole plus dasatinib-treated patients (Supplemental Table 2). However, in both arms, having PFS ≤ 6 months was significantly associated with co-expression of p-HER3 and p-Src (Supplemental Tables 3 and 4). AR expression correlated with expression of p-HER2, epidermal growth factor receptor (EGFR), p-EGFR, p-IGFR, p-ERK in letrozole plus dasatinib-treated patients who had PFS ≤ 6 months but not in letrozole-treated patients who had PFS ≤ 6 months.

## Discussion

This non-comparative phase-II trial in patients with HR-positive/HER2-negative MBC receiving their first AI in the metastatic setting demonstrated the primary endpoint, CBR, of 71% with letrozole plus dasatinib versus the projected CBR of 56% with letrozole plus dasatinib. The CBR rate of 66% in the letrozole-alone control arm also exceeded the assumed value of 39%. The higher than expected CBR with letrozole alone observed in this trial compared with the original first-line letrozole trial may have been due in part to the higher proportion of de novo MBC patients enrolled in this trial (37% vs 32% in Mouridsen et al).^15^ Recent phase-III trials combining first-line letrozole plus a CDK 4/6 inhibitor have shown CBRs of 70 to 73% with letrozole plus placebo, similar to the 66 and 71% CBRs observed with letrozole alone and with letrozole plus dasatinib in the current trial.^16,17^ Analysis of the secondary endpoint of PFS showed a longer than expected median PFS of 20.1 months with combined letrozole plus dasatinib. The median PFS of 9.9 months observed with letrozole alone is similar to the median PFS of 10 months reported with first-line letrozole in the Mouridsen pivotal trial.^15^ The long duration of PFS with letrozole/dasatinib could also have been seen because 42% of these patients had de novo metastatic disease and because 61% of patients were endocrine therapy-naïve.

The durable PFS observed with letrozole/dasatinib raises the hypothesis that dasatinib may slow the development of acquired resistance to letrozole. Results of two randomized phase-II studies which compared either exemestane or fulvestrant alone versus in combination with dasatinib showed no significant improvement in PFS with the addition of the Src inhibitor in patients with MBC that was resistant to a non-steroidal AI.^18,19^ It is possible that inhibiting Src can delay the remodeling of signaling pathways that lead to AI resistance in initially AI-sensitive breast cancers by inhibiting the formation of the ER/EGFR/Src complex.^20^ Also, in preclinical models, dasatinib increases ERα expression by inhibiting Src-mediated ERα degradation, and this may have contributed to the prolonged PFS observed with dasatinib plus letrozole.^7^

The AE profile observed with letrozole plus dasatinib in this study is consistent with the safety record of dasatinib in CML, as well as the AE results from the phase-II study in combination with exemestane.^18^ Overall, the combination was well tolerated, and although 26% of patients required dose reductions, the rate of grade 3/4 AEs, including pleural effusion, was low. No serious hepatotoxicity was observed, in contrast to results observed with another Src/Abl TKI, bosutinib. Two phase-II studies evaluated bosutinib in combination with first-line letrozole or second-line exemestane in postmenopausal patients with HR-positive/HER2-negative MBC.^21,22^ Although these combinations demonstrated anti-tumor activity, both trials were terminated early due to unfavorable safety profiles, including a 26–38% incidence of hepatotoxicity, and with approximately two-thirds of patients experiencing diarrhea.

**Fig. 3:**
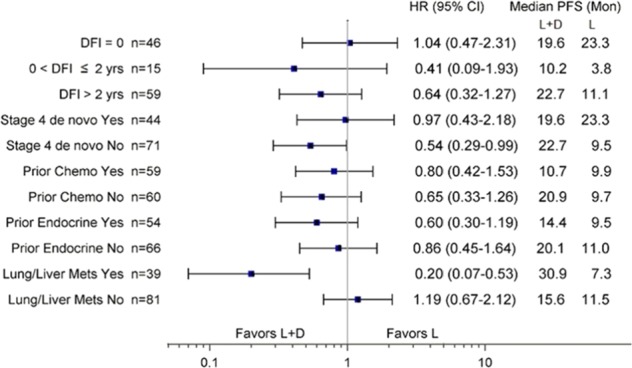
Forest plot of hazard ratios for progression-free survival by population subgroups

A secondary objective of this trial was to explore the impact of dasatinib on BMD assessed by DXA scan. In the dasatinib arm, the proportion of patients with osteopenia in the lumbar spine and/or hip was reduced from 32% at baseline to 14% on study treatment (Supplemental Fig. 1). It is plausible that dasatinib could improve bone density as Src kinase activity is essential to osteoclast development and function,^23,24^ and clinically achievable levels of dasatinib inhibit osteoclast activity.^13,14^ In preclinical studies in mice, low-dose dasatinib has been shown to promote osteoblast differentiation and matrix mineralization, and at the same time reduce osteoclast formation, differentiation, and resorption.^13^ The finding that some patients may have had improvement in their BMD on dasatinib is preliminary as DXA scans were missing in some patients, calibration of DXA imaging across centers was not performed, nor was central review of the scans (Fig. 3).

Our exploratory biomarker analyses in patients’ primary breast cancers failed to identify any strong predictors of benefit from dasatinib in the metastatic setting but did generate interesting hypotheses for future investigation. A limitation of these analyses is that some primary breast cancers may not harbor activated pathways that become manifest in established metastatic disease. Nonetheless, lack of clinical benefit with either letrozole or letrozole plus dasatinib (PFS ≤ 6 months) was associated with co-expression of p-Src and p-HER3 (and with co-expressed p-Src and EGFR in letrozole-treated patients) suggesting that coordinate activation of HER3 and Src may result in both letrozole and dasatinib resistance. The importance of HER3 signaling in endocrine resistance was recently demonstrated in a randomized phase-II study which showed a significant improvement in PFS when the HER3-targeted monoclonal antibody MM-121 was added to exemestane in patients with HR-positive MBC with high heregulin expression and low HER2 expression.^25^

AR expression in the primary breast cancers correlated with expression of p-HER2, EGFR, p-EGFR, p-IGFR, and p-ERK in patients who did not benefit from letrozole plus dasatinib (PFS ≤ 6 months), suggesting a potential interaction between AR and HER family receptors in letrozole/dasatinib resistance. Gene expression profiling has shown elevated AR and reduced ERα expression in tamoxifen-resistant breast cancers, while overexpression of AR can induce tamoxifen resistance in preclinical breast cancer models.^26^ These findings are hypothesis-generating due to the multiplicity of comparisons in this small sample set.

Moulder et al. utilized gene expression profiling on dasatinib-sensitive and -resistant cell lines to develop three potential predictive signatures of dasatinib sensitivity or resistance: a dasatinib sensitivity index, a Src pathway activity index, and a dasatinib target index.^27^ Among 133 breast cancer samples, the signatures identified distinct subsets that were predicted to be sensitive to dasatinib. However, because the patients had not been treated with dasatinib, no clinical correlations could be drawn. As there are not yet validated predictive biomarkers of response to dasatinib^25^, assessment of AR, p-Src and p-HER3 in future studies of dasatinib in ER + breast cancer would be of interest.

CDK 4/6 inhibitor therapy in combination with endocrine therapy is now the accepted standard of care for first-line treatment of ER + HER2– MBC due to substantially improved median PFS times of over 2 years compared to endocrine therapy alone. Src enables the emergence of acquired resistance to ER inhibition by forming a signaling complex between ER, EGFR, and Src.^20^ Because Src inhibition may retard acquired resistance to ER blockade, and because dasatinib is well tolerated over several years and the CBR of dasatinib plus letrozole is 71%, this agent could be a candidate “third leg” of a triplet combination with CDK 4/6 and ER inhibitors. Recent preclinical studies have demonstrated interactions between CDKs and Src in cell cycle regulation,^28,29^ and support investigating the addition of dasatinib to CDK 4/6 and ER inhibitors in breast cancer models.

The results of this non-comparative phase-II trial show a median PFS of 20.1 months with the Src inhibitor dasatinib plus letrozole in patients who had not previously received an AI for metastatic disease. The primary endpoint of CBR was 71% with letrozole plus dasatinib versus the projected CBR of the combination of 56%. The combination of dasatinib with letrozole was generally well tolerated, with a low rate of serious toxicities and some patients remaining on dasatinib longer than 2–3 years. Preliminary findings of improved BMD in some patients on dasatinib plus letrozole, and of AR and HER family expression in the primary breast cancers of patients who did not benefit from dasatinib plus letrozole could warrant further investigation.

## Methods

### Patient population

Eligible patients were postmenopausal women ≥18 years of age with histologically confirmed ER-positive (defined as >10% by immunohistochemistry), HER2-negative, unresectable, locally recurrent or MBC. Patients may have had either measurable or non-measurable (evaluable-only) disease, and could have received 0–1 prior chemotherapy regimens for MBC. Prior (neo)adjuvant chemotherapy was allowed. Prior adjuvant aromatase inhibitor (AI) was allowed if it had been stopped >1 year prior to study entry, but no prior AI therapy was permitted for MBC. Prior tamoxifen was allowed in either the adjuvant or metastatic settings. Intravenous (IV) bisphosphonates were not allowed during the first 8 weeks of study treatment. Other eligibility criteria included Eastern Cooperative Oncology Group (ECOG) performance status of 0–1; adequate hematologic, cardiac, renal, and liver, function. Patients with symptomatic central nervous system (CNS) metastases, or CNS metastases requiring steroid treatment or local therapy were not eligible. Patients with pleural or pericardial effusions of any grade were not eligible. The US Oncology Institutional Review Board (IRB) reviewed and approved the protocol. All participating patients signed the IRB-approved informed consent form.

### Study design

This was a multicenter, phase-II, randomized, non-comparative study (NCT00696072). Eligible patients were initially stratified by disease-free interval (DFI) from initial breast cancer diagnosis to first locally recurrent or MBC (≤2 years versus >2 years), and by prior tamoxifen for metastatic disease (yes/no). Patients were then randomized 1:1 to receive letrozole (2.5 mg orally daily) alone or with dasatinib (100 mg orally daily). Cycle length was defined as 28 days. Patients with disease progression on letrozole alone had the option of crossing over to receive dasatinib plus continued letrozole. Radiologic assessments to follow known disease were performed every 8 weeks, and bone scans were performed on all patients every 16 weeks (more frequently if disease progression suspected). Bone mineral density (BMD) assessment of lumbar spine and total hip by DXA scan was performed within 4 weeks prior to registration, and at the end of cycle 6. Bisphosphonate treatment was allowed after the first 8 weeks of study treatment at the discretion of the investigator.

### Study objectives

The primary objective of this study was to determine clinical benefit rate (CBR; complete response + partial response + stable disease ≥6 months) with letrozole plus dasatinib or letrozole monotherapy. Secondary objectives included overall response rates (ORR), median progression-free survival (PFS), 6- and 12-month PFS rates, overall survival (OS), changes in BMD from baseline to on-study assessment, safety, and evaluation of potential biomarkers to predict sensitivity and/or resistance to dasatinib.

### Safety assessment

Safety was assessed throughout the study. Adverse events (AEs) were graded and reported according to the National Cancer Institute Common Terminology Criteria for Adverse Events (version 3.0).

### Exploratory biomarker analysis

Formalin fixed paraffin embedded ((FFPE) primary breast cancer tissue was sent to a Clinical Laboratory Improvement Amendments (CLIA)-certified laboratory (Theranostics Health; Rockville, MD) for analysis by reverse phase protein array (RPPA). Sample protein lysates obtained from microdissected FFPE breast cancer tissues were printed onto slides. Samples were considered “acceptable” for analysis if the protein concentration of the undiluted sample following tumor enrichment and lysate preparation was ≥0.1 mg/mL. In addition, samples were eliminated prior to processing if the tumor content (percentage and total number of tumor cells within a section) of a particular sample was insufficient for processing. Immunostaining was carried out with 20 antibodies directed against HER pathway proteins, as well as AKT, S6 Ribosomal Protein, 4EBP1, ERK 1/2, IGFR1, MET, Src, MEK 1/2, STAT3, JAK-2, mTOR, Androgen Receptor (AR and phospho-AR) and the downstream target of Src, paxillin (see Supplemental Table 1 for specific antibody epitopes). Each protein biomarker was scored against the level of expression or activation (phosphorylation) across a population of breast cancers. Patient scores for each protein are based upon the number of standard deviations (SDs) from the population mean.

### Statistical analysis

Planned accrual was 60 patients per arm, to ensure at least 55 efficacy-evaluable patients per arm, for a total target accrual of 120 patients. This sample size provided 80% power to detect an absolute improvement in CBR from 39% with single agent letrozole to 56% with letrozole plus dasatinib, using a binomial test with 1-sided alpha level of 5%, allowing for a 10% rate of early drop-outs. The CBR of 39% with letrozole alone was extrapolated from the original first-line trial of letrozole where the CBR was 49%;^15^ we estimated a lower CBR in this trial assuming some adjuvant AI exposure in a proportion of patients. Because this was a randomized non-comparative study using a concurrent control arm, no statistical comparisons between the 2 arms are reported. The intent-to-treat (ITT) population included all patients registered on the study, and was used to describe the baseline demographics and disease characteristics, and for the PFS analyses. The evaluable population included all treated patients who met all inclusion and exclusion criteria and who received at least 1 dose of study drug, and was used for analysis of CBR and ORR. The safety population included all patients (eligible and ineligible) who received at least 1 dose of study drug, and was used in analyzing all safety parameters.

CBR was defined as the percentage of patients with complete response (CR), PR, and stable disease (SD) ≥ 6 months. ORR was defined as the percentage of CRs and PRs. The 95% confidence intervals (CI) were estimated for both ORR and CBR assuming binomial distribution. A Cochran–Mantel–Haenszel test was used to estimate the differences between ORRs and CBRs in the two arms after accounting for stratification factors. PFS was defined as time from date of registration to date of (first) progression or date of death. Patients without disease progression were censored at the last contact date. Kaplan–Meier methods were used to estimate PFS rates and median PFS using the intent-to-treat (ITT) population. Incidence and type of AEs were tabulated and summarized using descriptive statistics.

Student's *t*-test or Mann–Whitney U test and Spearman correlation coefficient (ρ) were used to assess biomarker association with PFS ≤6 months vs >6 months by treatment arm (unpaired 2 tails, *p* value). *P* values for the biomarker comparisons are exploratory, and have not been adjusted for multiplicity of comparisons.

### Reporting summary

Further information on research design is available in the Nature Research Reporting Summary linked to this article.

## Supplementary information

Supplementary Information

Reporting Summary Checklist

## Data Availability

The data generated and analyzed during this study are described in the following data record: 10.6084/m9.figshare.9792056.^30^ The datasets supporting the figures, tables and supplementary figures and tables in this published article are not publicly available in accordance with the policy of US Oncology Research, LLC, and the informed consents signed by the patients. Data supporting the figures, tables, and supplementary figures, can be accessed from the Vice President of US Oncology Research, Dr. Sandy Smith, on request, as described in the data record above. The data supporting the supplementary tables in the published article are not available from the US Oncology Research as these data were provided by the Clinical Laboratory Improvement Amendments (CLIA)-certified laboratory (Theranostics Health; Rockville, MD) that performed the experiments.^30^
