# Supplementary material for: Randomized phase-II evaluation of letrozole plus dasatinib in hormone receptor positive metastatic breast cancer patients
Source: NPJ Breast Cancer. 2019 Oct 28;5:36. doi: 10.1038/s41523-019-0132-8 (PMC6817898; doi:10.1038/s41523-019-0132-8)
Supplement: Supplementary file 1 — Supplementary Information [file 41523_2019_132_MOESM1_ESM.pdf]

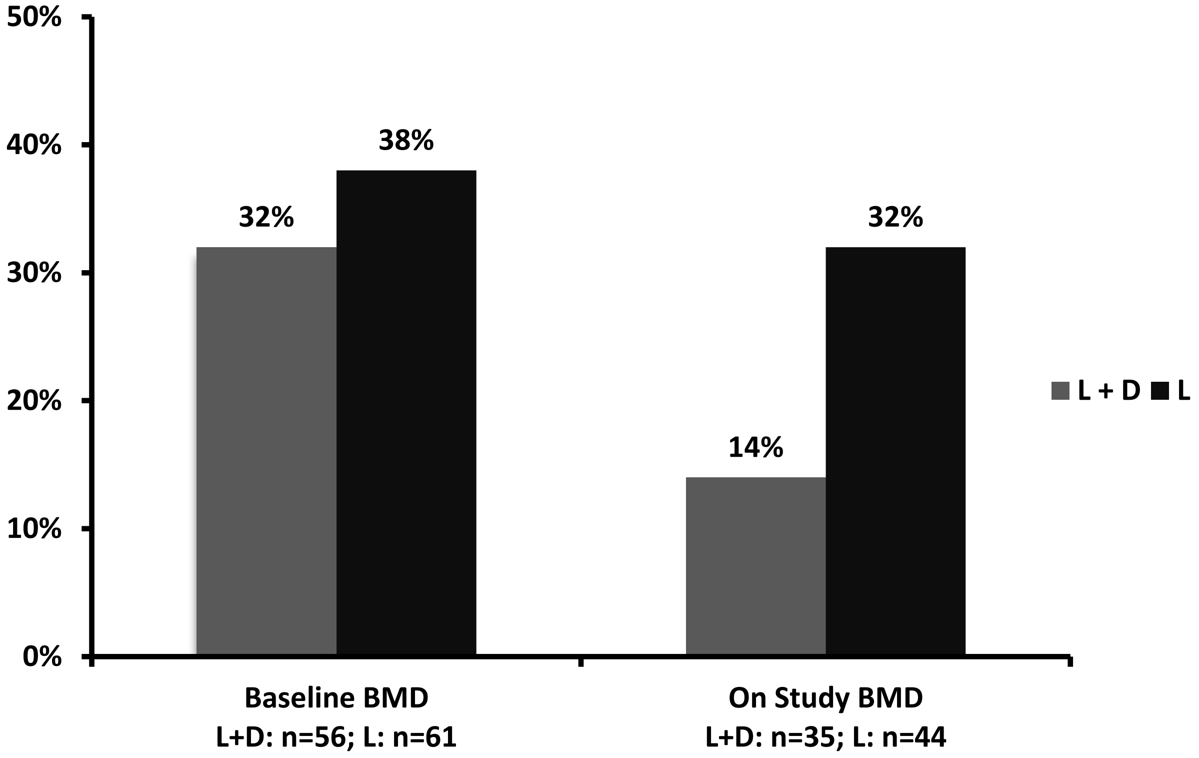

Supplemental Figure 1. Percentage of Patients with Bone Mineral Density T Scores <-1.5

Supplemental Table 1. Antibodies Used For Reverse Phase Protein Array

Antibody	Vendor	Catalog Number
EGFR (D38B1) XP	Cell Signaling	4267
EGFR (Y1068) (D7A5) XP	Cell Signaling	3777
ErbB2 Oncoprotein	Dako	A0485
ErbB2/HER2 (Y1248)	Novus Bio	NB100-81960
ErbB3/HER3 (1B2E)	Cell Signaling	4754
ErbB3/HER3 (Y1289) (21D3)	Cell Signaling	4791
Akt (S473) (D9E) XP	Cell Signaling	4060
mTOR (S2448)	Cell Signaling	2971
4E-BP1 (S65)	Cell Signaling	9451
S6 Ribo.Prot.(S235/236) (D57.2.2E) XP	Cell Signaling	4858
ERK 1/2 (T202/Y204)	Cell Signaling	9101
MEK1/2 (S217/221)	Cell Signaling	9121
Stat3 (Y705) (D3A7) XP	Cell Signaling	9145
Jak2 (Y1007/1008)	Cell Signaling	3771
IGF-IRb(Y1135/36)/IRb(Y1150/51)	Cell Signaling	3024
Src Family (Y416)	Cell Signaling	2101
Androgen Receptor (D6F11) XP RmAb	Cell Signaling	5153
Met (Y1234/1235) (D26) XP	Cell Signaling	3077
Androgen Receptor S650	Abcam	Ab47563
Paxillin (Y118)	Cell Signaling	2541

Supplemental Table 2. Overexpressed proteins differentiating PFS ≤ 6 months vs PFS > 6 months in letrozole- and letrozole plus dasatinib- treated patients.

	LETROZOLE					LETROZOLE + DASATINIB				
	≤6 months		>6 months			≤6 months		>6 months		
Markers	n	Mean (SD)	n	Mean (SD)	P value*	n	Mean (SD)	n	Mean (SD)	P value*
pS6 Ribo Prot.	9	0.35 (0.17)	13	0.34 (0.22)	0.76	6	0.39 (0.23)	12	0.31 (0.20)	0.41
pERBB2	8	0.33 (0.09)	11	0.25 (0.09)	0.07	5	0.31 (0.11)	13	0.25 (0.11)	0.31
EGFR	9	0.22 (0.06)	11	0.17 (0.10)	0.05	4	0.19 (0.02)	11	0.18 (0.05)	0.69
pEGFR	9	0.46 (0.08)	12	0.42 (0.14)	0.41	5	0.48 (0.12)	12	0.45 (0.12)	0.60
HER3	9	0.44 (0.10)	11	0.32 (0.09)	<0.01	4	0.39 (0.09)	12	0.36 (0.08)	0.55
pHER3	6	0.33 (0.08)	9	0.23 (0.08)	0.03	3	0.25 (0.17)	9	0.18 (0.10)	0.45
pAKT	9	0.27 (0.08)	12	0.24 (0.10)	0.58	5	0.27 (0.10)	11	0.25 (0.12)	0.72
p-mTOR	10	0.37 (0.20)	13	0.32 (0.18)	0.51	5	0.32 (0.10)	15	0.34 (0.16)	0.86
p4EBP1	9	0.56 (0.80)	15	0.39 (0.50)	0.39	5	0.24 (0.07)	12	0.21 (0.07)	0.42
pMEK	8	0.17 (0.05)	13	0.19 (0.09)	0.49	6	0.20 (0.13)	13	0.18 (0.11)	0.69
pERK	9	0.35 (0.25)	13	0.40 (0.25)	0.66	4	0.53 (0.40)	10	0.48 (0.31)	0.80
pJAK2	9	0.62 (0.17)	14	0.58 (0.21)	0.57	6	0.59 (0.17)	12	0.62 (0.22)	0.93
pSTAT3	10	0.70 (0.22)	14	0.68 (0.34)	0.41	6	0.65 (0.24)	13	0.67 (0.29)	0.93
pSRC	10	0.18 (0.10)	14	0.11 (0.05)	0.06	5	0.17 (0.15)	15	0.16 (0.09)	0.66
pIGFR	10	0.08 (0.02)	15	0.07 (0.01)	0.1	6	0.08 (0.02)	14	0.08 (0.02)	0.92
AR	11	0.25 (0.16)	15	0.29 (0.15)	0.58	6	0.19 (0.08)	15	0.23 (0.15)	0.82
pAR	10	0.30 (0.21)	14	0.27 (0.28)	0.52	5	0.27 (0.17)	13	0.36 (0.21)	0.38
pMET	10	0.13 (0.03)	15	0.11 (0.03)	0.05	6	0.14 (0.03)	14	0.13 (0.03)	0.76
pPaxillin	11	0.34 (0.18)	15	0.19 (0.11)	0.02	6	0.37 (0.19)	13	0.32 (0.18)	0.58
ERBB2	10	0.06 (0.01)	12	0.05 (0.03)	0.38	4	0.03 (0.02)	13	0.05 (0.03)	0.37

* P values are exploratory and have not been adjusted for multiplicity of comparisons.

Supplemental Table 3. Spearman rho correlations for patients treated with letrozole with PFS ≤6 months

	Letrozole		
	PFS ≤6 months		
Protein 1	Protein 2	Spearman Rho Correlation	P Value
ERBB2	pS6 Ribosomal Protein	0.930	0.008
pERBB2	HER3	0.802	0.040
pERBB2	pSTAT3	0.824	0.033
EGFR	pHER3	0.935	0.007
EGFR	p4E.BP1	0.993	0.000
EGFR	pSRC	0.949	0.005
EGFR	pIGFR	0.869	0.021
pEGFR	pAKT	0.964	0.003
pEGFR	pMEK1/2	0.963	0.003
pEGFR	pERK1/2	0.912	0.011
pEGFR	pJAK2	0.822	0.034
pEGFR	pIGFR	0.849	0.026
HER3	pMET	0.843	0.028
HER3	pPaxillin	0.830	0.031
pHER3	p4E.BP1	0.956	0.004
pHER3	pSRC	0.892	0.016
pHER3	pIGFR	0.981	0.001
pAKT	pMEK1/2	0.990	0.000
pAKT	pERK1/2	0.952	0.004
pAKT	pJAK2	0.896	0.015
pAKT	pSTAT3	0.776	0.049
pAKT	pIGFR	0.803	0.040
p4E.BP1	pSRC	0.935	0.007
p4E.BP1	pIGFR	0.906	0.013
pMEK1/2	pERK1/2	0.931	0.008
pMEK1/2	pJAK2	0.937	0.007
pMEK1/2	pSTAT3	0.843	0.028
pERK1/2	pJAK2	0.837	0.029
pJAK2	pSTAT3	0.970	0.002
pJAK2	pPaxillin	0.828	0.032
pSTAT3	pPaxillin	0.798	0.041
pSRC	pIGFR	0.796	0.042
pIGFR	pMET	0.802	0.040
pMET	pPaxillin	0.984	0.001

Supplemental Table 4. Spearman rho correlations for patients treated with letrozole + dasatinib with PFS ≤6 months

	Letrozole + Dasatinib		
	PFS ≤6 months		
Protein 1	Protein 2	Spearman Rho Correlation	P Value
pERBB2	EGFR	0.999	0.015
pERBB2	pEGFR	0.997	0.033
pERBB2	pERK1/2	0.999	0.020
pERBB2	pIGFR	0.998	0.027
pERBB2	Androgen Receptor	1.000	0.005
EGFR	pEGFR	0.999	0.019
EGFR	pERK1/2	0.997	0.035
EGFR	pIGFR	0.996	0.042
EGFR	Androgen Receptor	1.000	0.010
pEGFR	Androgen Receptor	0.998	0.028
pHER3	pMEK1/2	0.999	0.019
pHER3	pSRC	1.000	0.005
pMEK1/2	pSRC	0.999	0.024
pERK1/2	pIGFR	1.000	0.007
pERK1/2	Androgen Receptor	0.998	0.025
pJAK2	pSTAT3	0.998	0.026
pIGFR	Androgen Receptor	0.997	0.032
